# Influence of Dietary Intake on Sleeping Patterns of Medical Students

**DOI:** 10.7759/cureus.4106

**Published:** 2019-02-20

**Authors:** Maheen Nisar, Rubaab M Mohammad, Aleena Arshad, Irtiza Hashmi, Sarim M Yousuf, Saeeda Baig

**Affiliations:** 1 Biochemistry, Ziauddin University Hospital, Karachi, PAK

**Keywords:** melatonin, serotonin, tryptophan, sleep latency

## Abstract

Introduction

The aim of the study was to explore the association between the intake of specific food substances and the sleeping patterns of medical students.

Methods

A cross-sectional study was conducted with 440 medical students, aged 18-24 years, chosen through non-probability consecutive sampling from Karachi, Pakistan. The Pittsburgh sleep quality index (PSQI) was used to measure sleep quality and a self-made questionnaire that categorized foods according to their nutritional constitutions was used to measure the frequency of food consumption. Associations were evaluated using the chi-square test with the level of significance taken as p < 0.05 and strength of significance determined using Cramer’s V. logistic regression to predict good sleep quality.

Results

Significant associations were found with soybeans, whole grains, processed meats, leafy greens, dark chocolate, spices, dairy products, products high in fat and sugar, lima beans, and carbohydrates in relation to sleep quality, with soybeans exhibiting a particularly stronger relationship. The odds of good sleep quality were 2.5 times more likely with soybean intake, 3.26 times more likely with carbohydrates, and 6.57 times more likely with lima beans. Intake of papayas was associated with reduced sleep quality.

Conclusion

Intake of certain food substances has a significant association with sleep quality. Clinical trials focusing on the nutritional basis of these associations can lead to a new integrated focus on functional foods to combat poor sleep and sleep-related diseases.

## Introduction

Sleep deprivation is common amongst university students and medical students, in particular, are notorious for their poor sleeping habits. Decreased sleep duration, insomnia, and increased daytime sleepiness have been reported amongst medical students around the globe. Poor sleep quality has in turn been associated with impaired health and academic performance [1].

Although many psychological, biological, and social factors are known to affect sleep, diet has remained a relatively unappreciated determinant of sleep quality. Previous studies have established links between certain nutrients and sleep, for example, foods containing tryptophan, such as complex carbohydrates, raise melatonin levels in the body, a major hormone that regulates sleepiness [2]. Longer sleep has been found to be linked to increased consumption of selenium which is amply present in wholegrains, while a shorter sleeping duration has been linked with more lutein or zeaxanthin, such as found in green leafy vegetables [2-4]. However, even though previous studies have shown that functional foods can help prevent sleep disorders, hardly 1% of those affected are treated through functional foods [3].

This study was designed to explore the influence of the dietary intake of medical students on their sleep quality. Nutrients hypothesized in previous literature to influence sleep were selected and the frequency of their consumption was recorded from foods that made a popular part of Pakistani cuisine. Associations between the foods and overall sleep quality were recorded along with the strength of their impact on sleep quality. These associations were further examined according to the nutritional constituencies of the foods and their influence on neuroendocrinological pathways in the body.

## Materials and methods

Data was collected from students of all five years of medical study, using non-probability consecutive sampling, in accordance with their availability and consent. The duration of the study was three months, from November 2017 to January 2018, with data collected from three different medical schools of Karachi, Pakistan. Students with food allergies or chronic diseases or on any form of prolonged medical treatment were excluded from the study.

The major nutrients hypothesized in previous literature to influence sleep were selected and shortlisted according to their availability in foods popular in Pakistan. The frequency of consumption of those foods was then measured in medical students with a cross-sectional survey that was designed by following the format of a food frequency questionnaire developed by The Priority Research Centre for Physical Activity and Nutrition at the University of Newcastle [5]. Participants were asked to rate how much of a certain food they consumed in a week, by choosing between 'less than once a week or never' and 'once a week or more often'. Sleep quality was then assessed via the gold standard, Pittsburgh sleep quality index questionnaire (PSQI), with a global score of five or more indicative of poor sleep quality [6].

Data were entered into IBM Statistical Package for the Social Sciences 20.0 (SPSS 20, IBM, Armonk, NY, USA) and frequencies were calculated using descriptive statistics. Chi square test was used to find associations between the food categories and overall sleep quality, with p <0.05 taken as significant, and Cramer's V test was used to measure the strength of the associations. Predictions were made between different foods and good sleep quality using binominal logistic regression.

## Results

Out of the 440 students who participated in the study, 289 (65.7%) were females and 141 (32%) were males. Participants belonged to the age range 18-24 years (97%).

**Figure 1:**
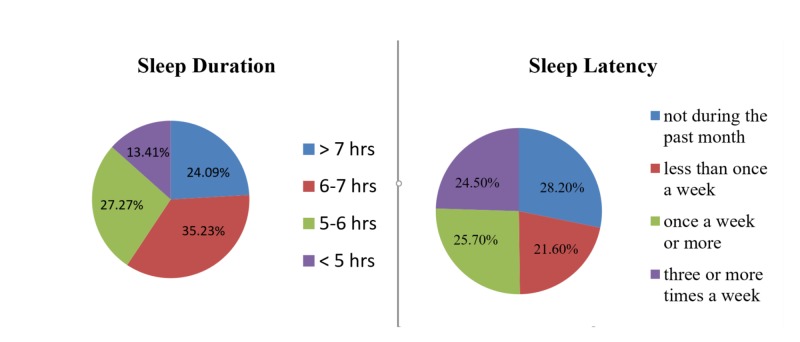
Sleep profile, showing sleep duration per night and frequency of difficulty sleeping past 30 minutes, of medical students in Karachi, Pakistan (n=440)

The sleep profile of our participants showed inadequate sleep for the majority of the students and a wide discrepancy with regards to sleep latency (Figure 1).

Only 24.1% of the participants received more than seven hours of sleep every night while 27.3% received five to six hours of sleep and 13.4% less than five hours of sleep each night. Frequency of sleep latency troubles varied widely, with 24.5% reporting difficulty falling asleep within 30 minutes of getting into bed, three or more times a week (Figure 1). This problem was faced by 25.7% of the students once or twice a week and 28.2% of them had no such trouble falling asleep for the past month. The time taken to fall asleep each night was 16 to 30 minutes for 31% of the students, while 31.1 % took less than 15 minutes. Subjective sleep quality was reported by the majority, 56.1%, as fairly good while 21.1 % and 7% reported it as very good and very bad respectively.

With a PSQI global score of less than five taken as good sleep quality (according to PSQI guidelines), associations were examined between the total sleep score of the students and the different foods. Table 1 shows the significant results of that analysis.

**Table 1:** Association of dietary intake with sleep quality using chi square test *p-value < 0.05 ^a^food intake less than once a week ^b^food intake once a week or more often ^1^PSQI score less than 5 ^2^PSQI score 5 or greater

Food		Total Sleep Score			
		Good Sleep Quality^1^	Poor Sleep Quality^2^	p-value	Cramer’s V
Soybeans (n=322)	Less^a^	167	110	<0.001*	0.352
	More^b^	19	26		
Whole grains (n=391)	Less	93	63	0.002*	0.279
	More	136	99		
Lima beans (n=338)	Less	188	124	0.045*	0.254
	More	10	16		
Spice (n=426)	Less	68	53	0.039*	0.227
	More	188	117		
Leafy greens (n=425)	Less	91	62	0.015*	0.242
	More	168	105		
Processed meats (n=429)	Less	136	84	0.006*	0.254
	More	121	88		
Dark chocolate (n=428)	Less	169	126	0.018*	0.239
	More	88	45		
Carbohydrates (n=427)	Less	34	16	0.047*	0.233
	More	224	153		
Fatty/sugary foods (n=425)	Less	94	57	0.044*	0.225
	More	158	116		
Dairy products (n=430)	Less	29	24	0.040*	0.259
	More	228	149		

With p-value <0.05 taken as significant, consequential associations were found with soybeans (p<0.001), whole grains (p=0.002), processed meats (p=0.006), leafy greens (p=0.015), dark chocolate (p=0.018), spices (p=0.039), dairy products (p=0.04), products high in fat and sugar (p=0.044), lima beans (p=0.045), and carbohydrates (p=0.047) in relation to sleep quality. A post-test Cramer’s V was done to determine the strengths of association, as shown in Table 1 and Table 2. While an appreciable level of significance was found with all the mentioned foods, soybeans (V=0.35) exhibited a particularly strong relationship with overall sleep quality.

Logistic regression analysis was performed to ascertain the effects of different foods on the likelihood of participants having good sleep quality (Table 2). The logistic regression model was statistically significant, χ2(11) = 28.149, p = 0.003. The model explained 12.8% (Nagelkerke R2) of the variance in sleep quality and correctly classified 63.3% of cases.

**Table 2:** Binomial logistic regression results of experiencing good sleep quality among medical students, in Karachi, Pakistan *p-value < 0.05 B = beta, S.E. = standard error, D.F. = degrees of freedom , Sig. = significance level, Exp(B) = exponentiation of the B coefficient, CI = confidence interval

Predictors	B	S.E.	Wald	D.F	Sig.	Exp(B)	95 % CI for Exp(B)	
							Lower	Upper
Whole grains	-.200	.275	.528	1	.467	.819	.477	1.404
Soybeans	.922	.410	5.060	1	.024*	2.514	1.126	5.614
Lima beans	1.883	.716	6.919	1	.009*	6.572	1.616	26.726
Papaya	-.929	.389	5.701	1	.017*	.395	.184	.847
Fatty/sugary foods	-.131	.286	.210	1	.647	.877	.500	1.537
Dairy products	-.844	.529	2.545	1	.111	.430	.153	1.213
Leafy greens	.236	.285	.685	1	.408	1.266	.724	2.213
Spice	-.549	.310	3.148	1	.076	.577	.315	1.059
Processed meat	.353	.275	1.644	1	.200	1.423	.830	2.441
Dark chocolate	-.339	.295	1.325	1	.250	.712	.400	1.269
Carbohydrates	1.181	.545	4.689	1	.030*	3.258	1.119	9.490
Constant	-1.655	.798	4.307	1	.038	.191	.477	1.404

Significant associations were found between sleep quality and intake of soybeans (p=0.024), lima beans (p=0.009), papaya (p=0.017), and carbohydrates (p=0.03). The odds of good sleep quality were 2.5 times more likely with soybean intake (OR=2.51; CI 1.13 - 5.61), 3.26 times more likely with carbohydrates (OR=3.26; CI 1.12 – 9.49) and 6.57 times more likely with lima beans (OR=6.57; CI 1.62 – 26.7). Intake of papayas was associated with reduced sleep quality (OR=0.40; CI 0.18 – 0.85).

## Discussion

This study demonstrated that intake of certain food substances had a significant association with sleep quality. Most of these foods affected the brain transmitter serotonin, which helps regulate the body’s sleep wake cycle and internal clock. Most notably tryptophan, a precursor to serotonin and hormone melatonin, plays a dominant role in the biochemistry of sleep-inducing foods [7].

Soybeans were found to have the strongest association with sleep quality (V = 0.35). A class of phytoestrogens called isoflavones, similar to human estrogen, are found in soya beans. Since estrogen modulates sleep duration and quality, it is hypothesized that isoflavones benefit sleep in a similar way [8]. Tryptophan and complex carbohydrates which influence serotonin production are also constituents of soybeans. The presence of L-ornithine may also enhance sleep quality by reducing stress and anxiety. In a study on L-ornithine, it was concluded that its supplementation has the potential to relieve stress and improve sleep quality related to fatigue both objectively and subjectively [9].

Whole grains, which strongly influenced overall sleep quality (V=0.28), are enriched with a range of nutrients known to impact sleep. Magnesium works as a natural muscle relaxant by binding to gamma-aminobutyric acid (GABA) receptors and also guides the sleep-wake cycles in our body by regulating melatonin. The presence of butyric acid in whole grains also help the body to produce GABA [3]. The complex carbohydrates raise serotonin and lower the stress hormone, cortisol, high levels of which inhibit sleep [10]. Whole grains are also rich in tryptophan and, according to the National Sleep Foundation (NSF), carbohydrates help deliver tryptophan to the brain [11]. The insulin spike caused after the carbohydrate intake reduces amino acid levels in blood, enabling tryptophan to cross the blood-brain barrier for conversion to serotonin. They also contain the antioxidant selenium that helps settle restlessness/irritability at bedtime and restores the body’s internal equilibrium during sleep to foster a calmer state [3].

Leafy greens (V=0.24), are enriched with micronutrients which may also influence sleep, such as tryptophan, potassium, magnesium, fiber, iron, calcium, vitamin C, lutein and zeaxanthin, choline, complex carbs, and beta carotene [12]. Fiber has been shown to be associated with deeper and more restorative sleep. Lutein and zeaxanthin, also known as the macular carotenoids, are natural filters of high-energy blue light which is known to suppress melatonin production [13]. Other carotenoids like lycopene and beta carotene, antioxidants with effects on cell differentiation and growth, have also been associated with less difficulty falling asleep [12].

A moderately strong level of association was seen between dairy products and sleep quality (V=0.26). Rich in both calcium and tryptophan, calcium helps the brain use the tryptophan to manufacture melatonin [13]. Dairy products are also a good source of the mineral selenium, whose antioxidant properties are linked with improved sleep [3]. Butanoic acid, found in cow milk, has been found to be associated with a decreased likelihood of difficulty maintaining sleep [13]. Cheeses contain the sleep promoter serotonin and low-fat yogurts contain magnesium, a muscle relaxant [3].

White rice (V=0.31) and jasmine rice (V=0.18) containing magnesium, phosphorus, manganese, selenium, iron, folic acid, thiamine, and niacin, showed a stronger and weaker association with sleep latency, respectively. Other than the carbohydrate content increasing tryptophan levels, a lower serum folic level is associated with a shorter sleep duration. Folate and vitamin B-12 convert homocysteine to methionine, thereby improving DNA-methylation patterns associated with circadian rhythmicity and melatonin metabolism [14]. Jasmine rice has a higher glycemic index (GI) than white rice, and it was concluded in a study by the University of Sydney that the high GI jasmine rice provided more insulin to increase tryptophan influx into the participants' brains [15]. Our results contradict this study by showing white rice to be more effective. One reason hypothesized for this discrepancy is that rice is more popular as a supporting dish in Pakistani culture, usually with spicy meals like curries. The negative effect of the spices on sleep latency may override the positive effects of the rice. Spicy foods (V=0.23) are notorious for causing heartburn, indigestion, and acid reflux. Heartburn can be made worse while sleeping, as lying down allows the acids to creep up into the esophagus and burn the sensitive lining. It has also been noted that an intake of spicy meals elevates body temperature, which has been linked in other studies to poorer sleep quality [16].

 Lima beans contain high levels of phosphorous, magnesium, L-ornithine, orexin and B6, with phosphorous especially crucial for cellular repair, energy metabolism, and sleep. Low levels of phosphorous have been associated with sleep deficit and phosphorous supplementation has been shown to increase sleep efficiency and reduce episodes of waking after sleep onset [2].

Dark chocolate may have a positive or negative effect on sleep (V=0.24), depending on its time of intake. The NSF recommends avoiding chocolate before bedtime as it contains caffeine and theobromine -- both stimulants of the central nervous system. Theobromine is also a cardiac stimulant, that negatively affects sleep [17]. However, because of its magnesium concentration, dark chocolate could help induce sleep as well [3, 18].

Higher fat intake has been associated with sleep disorders (V=0.23). Studies have shown that people with high consumption of fat and sugar have shorter sleeping durations, lighter, less restorative sleep, with more awakenings throughout the night [3]. Clinical intervention studies showed greater fat intake during periods of sleep restriction compared with during normal habitual sleep. Fat especially was highlighted as a macronutrient of choice during sleep restrictive periods [19]. It has been demonstrated in several studies that high sugar intake decreases orexin cell activity and that a similar decrease in orexin sensitivity is brought about with weight gain from high-fat foods. This leads to poor and interrupted sleep, orexin being a neurochemical critical in regulating the sleep-wake cycle and managing appetite and particularly involved in stimulating wakefulness and creating the urge to eat [20]. A lack of orexin is believed to be the primary cause of narcolepsy. People with narcolepsy experience extreme levels of daytime sleepiness and “sleep attacks,” when they may fall asleep abruptly at any time during the day [21].

Herbal teas are popularly used as sedatives to calm nerves and reduce anxiety. They can be used to treat hysteria, nightmares, insomnia, and other sleep problems (V=0.25). L-Threonine, an amino acid found in tea leaves, and especially present in green tea, binds to GABA receptors and induces changes in brain waves indicative of relaxation. It has been shown to improve sleep quality in children with attention deficit hyperactivity disorder (ADHD) [22]. Apigenin, a component of chamomile, possesses anxiolytic and sedative effects. A study suggested that apigenin augmented pentobarbital induced sleep behaviors through chloride ion channel activation [23]. Components of magnolia bark enhance the activity of GABA receptors in the brain and some herbs like rhodiola rosea contain 140 known bioactive compounds, many of which are effective against depression, anxiety, fatigue, and stress [24].

Papayas contain vitamin C, folate, vitamin A, magnesium, potassium, and copper which may play a part in their association with sleep quality (V=0.24). Micronutrients may be associated with antagonists of excitatory transmissions through the N-methyl-D-aspartate receptors (NDMA) and dopaminergic neurons, and may also potentiate inhibitory transmissions, such as GABA receptors [25]. Their retinoic acid content, a metabolite of vitamin A, significantly up-regulates the expression of clock/bmal dependent circadian genes, thus modulating the circadian-sleep regulatory process of sleep stages [26]. 

Most of the past studies conducted to investigate connections between diet and sleep primarily explore total calorie intake in association with sleep. Some studies categorize food according to healthy and unhealthy nourishment, while a few others divide foods according to carbohydrates, proteins, and fats. Only limited progress has been made on classifying specific dietary nutrients according to their sleep enhancing or deteriorative characteristics. Comparatively exhaustive studies on dietary intake are still missing certain influential and important nutrients, such as vitamin B6 [3]. Furthermore, most studies of this nature have been conducted in the United States, leaving the evaluation of the nutrition of populations in Pakistan largely unexplored.

Medical students are notorious for their poor sleeping habits, and the results of our study conformed with that previously established trend [1]. The optimal sleep duration for ages 18-64 years, as stated by the NSF, is seven to nine hours. However, a concerning 40.7% of our subjects got less than six hours of sleep per night. Nutrition and lifestyle education are not a prioritized part of the medical curriculum. The application of nutrition science to clinical practice is often neglected, and there is a poor collaboration with nutrition professionals [27]. Health professionals should be made aware of the relationship between diet and sleep, and future research should focus on more clinical trials to explore the causal relationship between promising dietary foods and sleep. This will help in developing more comprehensive guidelines for new and integrative therapies directed towards battling sleeping disorders. Pakistan being a low income and an agricultural country, this new integrated focus on the potential of Pakistani food to combat poor sleep and sleep-related diseases will be economical and convenient, as well as a superior replacement for the increasing dependence on sleeping pills, caffeine and alcohol [28]. Finally, further research in this area would empower populations at high risk for sleeping disorders to make active decisions towards improving their own lifestyle. 

This study has potential limitations. Although an effort was made to emphasize the role of diet on sleep quality by excluding students with chronic diseases or food allergies and students on prolonged medications, the complex nature of sleep suggests other determinants may have influenced results. These include social factors such as stress or anxiety, behavioral factors such as excess light exposure from electronics, and biological factors such as pain from recent injuries. Clinical trials rather than cross-sectional studies are further recommended as more effective study designs towards establishing connections between nutrients and sleep quality. 

## Conclusions

Poor sleep quality is prevalent among medical students, with our study showing almost half the students sleeping less than the amount recommended by the NSF. As suggested by medical literature, while sleeping pills and energy drinks are popular among students, dietary modifications that influence sleep, and nutrition science in general, are an underappreciated aspect of clinical medicine. Intake of certain food substances has a significant strength of association with sleep quality, as demonstrated by our data, with foods like soybeans, lima beans and carbohydrates exhibiting a particularly positive relationship. Clinical trials focusing on the chemical basis of these associations can lead to a new integrated focus on functional foods to combat poor sleep and sleep-related diseases.
